# Association of celiac disease with menstrual and hormonal disturbances: a systematic review and meta-analysis

**DOI:** 10.1186/s12958-026-01572-7

**Published:** 2026-06-22

**Authors:** Michail Delis, Elpida Emmanouilidou-Fotoulaki, Eirini Iordanidou, Achilleas G. Tsimperidis, Olga Giouleme, Dimitrios G. Goulis, Theodoros Theodoridis

**Affiliations:** 1 1 st Department of Obstetrics and Gynecology, Medical School, Aristotle University of Thessaloniki, “Papageorgiou” General Hospital, Thessaloniki, Greece; 2 1 st Department of Pediatrics, Medical School, Aristotle University of Thessaloniki, “Hippokration” General Hospital, Thessaloniki, Greece; 3 Gastroenterology and Hepatology Division, 2nd Propedeutic Department of Internal Medicine, Medical School, Aristotle University of Thessaloniki, “Hippokration” General Hospital, Thessaloniki, Greece

**Keywords:** Celiac disease, Menstrual, Menarche, Menopause, Hormonal, Ovarian

## Abstract

**Background:**

Disturbances in the female reproductive system are known extraintestinal manifestations of celiac disease (CD). However, the reliability of the existing literature is limited by small study populations and heterogeneous study designs. This study is the first systematic review and meta-analysis to synthesize all available evidence and provide a comprehensive evaluation of the association between CD and female menstrual and hormonal parameters.

**Methods:**

A systematic literature search was conducted in PubMed, Scopus, and Cochrane Central databases using keywords related to CD, menstrual characteristics, and hormonal markers. Studies published until August 2025 were included.

**Results:**

Our analysis demonstrated a significantly higher mean age at menarche in girls with CD compared with controls [pooled mean difference (MD): 0.64 years, 95% confidence interval (CI): 0.32 to 0.95, *p* = 0.0007), as well as an increased risk of abnormal uterine bleeding [pooled odds ratio (OR): 2.11, 95% CI: 1.23 to 3.63, *p* < 0.05] and amenorrhea (pooled OR: 4.03, 95% CI: 1.11 to 14.65, *p* = 0.0394). Menopausal age was not found to be earlier in women with CD (pooled MD: -1.31 years, 95% CI: -3.02 to 0.40, *p* = 0.1053). Adherence to a gluten-free diet was not associated with age at menarche when adherent and non-adherent patients were compared (pooled MD: 0.56 years, 95% CI: -0.72 to 1.83, *p* = 0.260). Furthermore, none of the evaluated biochemical hormonal markers (FSH, LH, estradiol, prolactin, and AMH) differed significantly between women with CD and controls.

**Conclusion:**

This systematic review and meta-analysis reinforces the existing evidence that CD may adversely affect several aspects of menstrual health in women without significantly impacting biochemical hormonal markers.

**Supplementary Information:**

The online version contains supplementary material available at https://doi.org/10.1186/s12958-026-01572-7.

## Background

Celiac disease (CD) is a chronic autoimmune disease characterized by intestinal mucosal damage, affecting genetically predisposed individuals upon exposure to gluten [1]. If left untreated, multiple organ systems can be impaired in affected individuals [2]. The only known treatment is lifelong gluten abstinence [gluten-free diet (GFD)] [3]. The consequences of CD on women’s reproductive health have been investigated in recent decades, including potential gestational, menstrual, and fertility complications [4–7].

Although the effects of CD on the reproductive system are established extraintestinal manifestations, the existing literature seems controversial in many aspects [8, 9]. Understanding the intricate association between CD and female reproductive health is crucial for improving the clinical management and quality of life of affected women in the future. However, the heterogeneity of the study designs and outcome measurements has limited the ability to draw definitive conclusions.

This is the first attempt to combine data on women with CD menstrual characteristics and biochemical hormonal markers, synthesizing them in a meta-analysis to obtain a cumulative result by combining all the current bibliography.

## Methods

### Search strategy and study selection

This systematic review is registered in the Open Science Framework (OSF) (https://doi.org/10.17605/OSF.IO/FV3Y6), and a full protocol was submitted according to the PRISMA guidelines [10]. The search strategy aimed to identify studies presenting data related to menstrual characteristics and hormonal markers in women with CD, and comparing them with those of healthy controls.

A systematic review was conducted in the MEDLINE, SCOPUS, and CENTRAL libraries until 03/08/2025. The keywords used were “celiac disease”, “menstrual”, “menarche”,”menopause”, “ovarian reserve”, “AMH”, and “FSH”. The exact search queries are shown in the registered protocol. The reference lists of the full-text screened articles and relevant reviews were also screened manually for relevant literature. Prospective and retrospective case–control studies or cohorts with a control arm were included in this review. Only studies on humans and papers in English were included. Duplicate removal, as well as title, abstract, and full-text screening, was independently performed by two authors (M.D. and E.E.F.) using the Rayyan AI tool [11]. Disagreements were resolved through discussion or, if agreement could not be reached, by consulting another reviewer (T.T.).

### Data analysis and quality assessment

#### Data extraction and conversion

The data of the included studies were independently extracted by two authors (M.D. and E.E.F.) and recorded on predetermined sheets. Authors, date, study type, menstrual characteristics such as age at menarche and menopause and menstrual disorders, as well as hormonal markers and ovarian reserve markers were the data of interest for synthesis. Other characteristics of the study population, such as age, CD diagnosis method, and dietary period, were also noted. The authors of the studies were not contacted, and when the study data were not extractable, they were left out of the synthesis as “not extractable data”. For continuous outcomes, mean values and standard deviations (SD) were extracted from the included studies and used for quantitative data analysis. Two studies [12, 13] were not included in the quantitative synthesis because they only reported mean values without any measures of variability. For studies reporting medians with interquartile ranges [14, 15], we applied the method described by Wan et al. [16]. For studies reporting medians with ranges [6, 17], we applied the approach described by Hozo et al. [18]. Both conversion methods were implemented in R using the ‘estmeansd’ package. For studies reporting highly skewed data (median substantially lower than the estimated mean), we acknowledge that the conversion methods may overestimate the mean and standard deviation; thus, serum estradiol (E₂) concentrations from one study [14] were excluded from the quantitative synthesis due to highly skewed distributions that precluded reliable conversion from median to mean. Sensitivity analyses were performed to assess the impact of including these studies in the meta-analysis.

#### Handling of multiple subgroups

When studies reported data for two mutually exclusive subgroups (e.g., treated and untreated patients, different age groups) [9, 19, 20], we combined these into a single group using pooled means and standard deviations, calculated according to methods described in the Cochrane Handbook for Systematic Reviews of Interventions (Section 6.5.2.10) [21]. For outcomes in which individual studies reported multiple (more than two) age-stratified subgroups, we used robust variance estimation (RVE) with cluster-robust standard errors to account for the dependence between effect sizes from the same study [19, 22]. A within-study correlation (ρ) of 0.8 between subgroups was assumed, consistent with the recommendations for meta-analyses of dependent effect sizes. Sensitivity analyses were conducted using alternative correlation assumptions (ρ = 0.6 and ρ = 0.95) [21].

#### Effect size calculation

For continuous outcomes, mean differences (MD) with 95% confidence intervals (CI) were calculated. For dichotomous outcomes, odds ratios (OR) with 95% confidence intervals (CI) were calculated. Studies with zero events in both groups [23] were excluded (for amenorrhea) because they provided no information on the treatment effects. For studies with zero events in one group, a continuity correction of 0.5 was applied to all the cells.

#### Meta-analysis models

A random-effects meta-analysis was performed using the restricted maximum likelihood (REML) method to account for between-study heterogeneity. For outcomes with multiple dependent effect sizes per study, we employed the RVE using a correlated-effects model with small-sample corrections. Between-study heterogeneity was quantified using the I^2^ statistic and τ^2^ (tau-squared). Heterogeneity was interpreted as low (I^2^ < 25%), moderate (25% ≤ I^2^ < 50%), substantial (50% ≤ I^2^ < 75%), or considerable (I^2^ ≥ 75%). A leave-one-out sensitivity analysis was conducted by sequentially removing each study and recalculating the pooled estimates to identify influential studies. Sensitivity to the assumed within-study correlation (ρ) was examined by repeating the RVE analyses with different correlation values. All analyses were conducted using R version 4.2.3 with the ‘meta’,’metafor’, and ‘robumeta’ packages.

#### Quality assessment

Quality assessment of all included studies was conducted individually by two authors (M.D. and E.E.F.) using the Newcastle–Ottawa Risk of Bias tool. Discrepancies were resolved through discussion between the reviewers, and the results are presented in Table 1. A graphical presentation of the results is provided in the Supplementary Material.

## Results

**Fig. 1:**
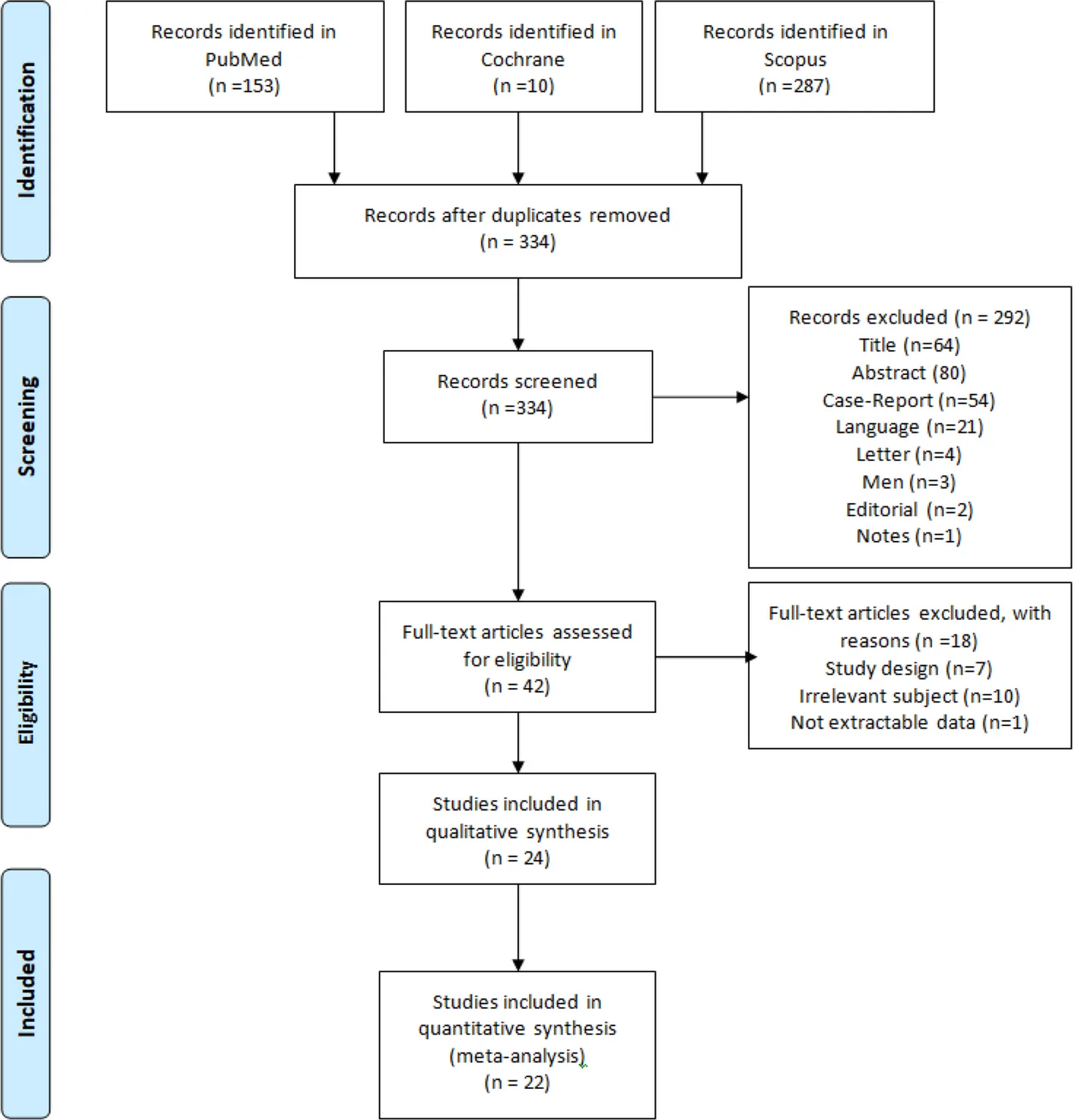
Study PRISMA flowchart

The search yielded 334 original citations after duplicate removal (Fig. 1). After removing case reports, non-English language articles, letters to the editor, notes, men studies, editorials, titles, and abstracts, 42 papers were eligible for full-text screening. After excluding seven studies with non-acceptable study designs [34–40], ten studies with irrelevant subjects [41–50] and one study without extractable data [51], 24 studies were included in the final analysis. The characteristics of all the included studies are presented in Table 1.

**Table 1:** Characteristics of all included studies

Study	Design	Participants	Measurements	Exclusion Criteria	Risk of Bias (NOS)
Ali 2024 [24]	Cross- sectional Case–control	Cases: 89 women with CD Controls: 93 healthy female subjects Age: 25.4 ± 4.9 years CD diagnosis criteria: N/S	Anthropometric, Estrogen, Progesterone, AMH, FSH, IL-10, CD antibodies	None	Selection 3/4 Comparability 1/2 Exposure 2/3 Sum 6/9
Bayrak 2020 [25]	Cross- sectional Case–control	Cases: 228 patients with CD Controls: 135 non-CD healthy individuals 65.2% female Age: 8–18 years CD diagnosis criteria: ESPGHAN	Anthropometric, Tanner stage Age at menarche, Transferrin saturation, Total IBC, Vitamin D, FSH, LH, E_2_, Testosterone, Prolactin	Type 1 diabetes, Down syndrome Hypothyroidism, Selective IgA deficiency Chronic renal disease, Cardiac diseases Hepatic disease, Neurologic disease	Selection 4/4 Comparability 2/2 Exposure 3/3 Sum 9/9
Cakmak 2017 [26]	Cross- sectional Case–Control	Cases: 46 women with CD Controls: 40 healthy female subjects Age: 18–45 years CD diagnosis criteria: ACG guidelines	Anthropometric, FSH, LH, E_2_, PRL, AMH, Ovarian volume, AFC	Smoking, Premature menopause Chronic renal disease, Chronic hepatic disease, Malignancy, History of infertility Hormonotherapy, Pregnancy History of surgery, PCOS	Selection 4/4 Comparability 2/2 Exposure 2/3 Sum 8/9
Comba 2020 [27]	Cross- sectional Case–Control	Cases 21: Adolescent girls with CD Controls: 24 age-matched, healthy subjects with regular menstrual periods Age: 13–18 years CD diagnosis criteria: ESPGHAN	Anthropometric, FSH, LH, E_2_, PRL, AMH, AFC, Ovarian volume	One year since menarche Obesity or malnutrition Hyperandrogenism symptoms Findings of hyperthyroidism or hypothyroidism Hyperprolactinemia, Chronic diseases Hormonal contraceptives Delayed menarche, Early puberty Delayed puberty, Other sexual disorder	Selection 4/4 Comparability 2/2 Exposure 2/3 Sum 8/9
Ehsani-Ardakani 2014 [28]	Prospective Case–Control	Cases: 50 women with CD diagnosed for the first time Controls: 70 healthy women Age: 18–45 years CD diagnosis criteria: positive serology and confirmation by histological assessment of small bowel biopsies	Anthropometric, Infertility, AUB, Abortions	Pathology contributed in AUB Abnormal TSH or Prolactin Systematic disease	Selection 3/4 Comparability 2/2 Exposure 2/3 Sum 7/9
Ferguson 1982 [4]	Cross- sectional Case–Control	74 CD women divided according to GFD adherence CD diagnosis criteria: N/S	Menstrual history, Age at menarche Age at menopause, Time for conception, Pregnancy outcome	None	Selection 3/4 Comparability 0/2 Exposure 2/3 Sum 5/9
Juneau 2018 [15]	Prospective cohort	Population: 995 women undergoing IVF Cases: 28 of them tested positive for CD antibodies Age: 18–45 years CD diagnosis criteria: At least one of tTG/EMA positive	BMI, AMH, IVF parameters, Pregnancy outcomes	Couples with a sole diagnosis of male-factor infertility Couples who were using donor gametes or a gestational carrier	Selection 4/4 Comparability 2/2 Exposure 3/3 Sum 9/9
Kotze 2004 [19]	Cross- sectional Case–Control	Cases: 76 adult and 18 adolescents with CD were analyzed according to their nutritional status and adherence to a gluten-free diet Controls: 84 adults and 22 adolescents with irritable bowel syndrome Age: 12–78 years CD diagnosis criteria: histological assessment of small bowel biopsies	Somatometric, Gluten diet adherence Menstrual history, Age at menarche, Age at menopause, Miscarriages Infertility, Anemia, Albumin	Women without an active sex life, using contraceptive methods, menopause	Selection 4/4 Comparability 1/2 Exposure 3/3 Sum 8/9
Kotze 2020 [29]	Retrospective Case–control	Cases: 214 women with CD Controls: 286 women Age: 18–72 years CD diagnosis criteria: histological assessment of small bowel biopsies	Age of menarche Age at menopause Number of pregnancies Miscarriages	Other diseases that might interfere with the fertility Chronic inflammatory diseases	Selection 4/4 Comparability 1/2 Exposure 3/3 Sum 8/9
Mahmoudi 2025 [30]	Cross-sectional Case–control	Cases: 27 women with CD Controls: 27 healthy women Age: 18–45 years CD diagnosis criteria: ACG guidelines	Anthropometric, FSH, LH, PRL, TSH, AMH, AFC, Ovarian volume Pregnancy complications Miscarriages, Age at menarche	Smoking, Premature menopause Chronic renal disease Chronic hepatic disease Malignancy, History of infertility Hormone therapy, Pregnancy History of surgery, PCOS Irregular menstruation	Selection 4/4 Comparability 2/2 Exposure 3/3 Sum 9/9
Martinelli 2010[31]	Cross-sectional Case–control	Cases: 62 women with CD Controls: 186 healthy women Age 17–49 years CD diagnosis criteria: serum concentrations of anti-gliadin and/or anti endomysial, and/or anti-transglutamminase antibodies and/or small intestine biopsy	Menstrual history Age at menarche Pregnancy complications	None	Selection 4/4 Comparability 2/2 Exposure 3/3 Sum 9/9
Micetic-Turk 2018 [8]	Cross-sectional Case–control	Cases: 145 CD patients Age 15–51 years Controls: 162 healthy women Age 18–55 years CD diagnosis criteria: ESPGHAN	Menstrual history	None	Selection 4/4 Comparability 2/2 Exposure 3/3 Sum 9/9
Moleski 2015 [12]	Retrospectivecohort	Cases:329 CD patients biopsy diagnosed Controls: 641 patients with no history of CD CD diagnosis criteria: histological assessment of small bowel biopsies	Age at menarche, Age at menopause Infertility, Miscarriages, Pregnancy complications	Not biopsy diagnosed	Selection 4/4 Comparability 1/2 Exposure 3/3 Sum 8/9
Molteni 1990 [6]	Cross-sectional Case–control	Cases: 54 women with CD Controls: 54 healthy women Age 16–62 years CD diagnosis criteria: histological assessment of small bowel biopsies and respond to GF diet	Menstrual history, Age at menarche Miscarriages, Infertility	None	Selection 4/4 Comparability 1/2 Exposure 3/3 Sum 8/9
Nanah 2025 [22]	Database Case–control	Cases: 9,368 patients with CD Controls: 25,771,736 without CD Divided in 4 groups Age: 10–60 years CD diagnosis criteria: (K90.0) registry + one or more of: Dermatitis Herpetiformis (L13.0)/histological findings from digestive organs (R85.7)/dietary counselling and surveillance (Z71.3), ≥ 30 U/mL of tTG (IgA) antibody or deamidated gliadin peptide, or a prior positive result of EMA IgA titre	Primary ovarian failure PCOS, Menstrual history Infertility, Recurrent pregnancy loss Miscarriages, Premature births Endometriosis, Menopausal disorders	Νon-celiac gluten sensitivity Patients seen > 20 years ago	Selection 4/4 Comparability 2/2 Exposure 3/3 Sum 9/9
Pogacar 2019 [32]	Retrospectivecase-control	Cases: 144 women with CD Controls: 71 healthy women CD diagnosis criteria: histological assessment of small bowel biopsies	Age at menarche, Infertility Miscarriages, Pregnancy complications	Gastrointestinal disorders	Selection 3/4 Comparability 2/2 Exposure 2/3 Sum 7/9
Prasad 2024 [33]	Cross-sectional Case–control	Cases: 288 CD women Controls: 586 healthy women Age: 21–40 years CD diagnosis criteria: clinical symptoms, a positive IgA anti-tTG and presence of villous abnormalities of modified Marsh grade 2 or more on duodenal biopsy	Menstrual history, Age at menarche Age at menarche, Miscarriages, Infertility, Pregnancy complications Marriage status	None	Selection 4/4 Comparability 1/2 Exposure 3/3 Sum 8/9
Santonicola 2011 [9]	Cross-sectional Case–control	Cases: • 33 CD menopausal (untreated CD group) • 25 CD on GFD at least 10 years before menopause (treated CD group) Controls: 45 healthy women Age: menopausal CD diagnosis criteria: small bowel biopsy and search for CD-specific serum antibodies	Anthropometric Age at menarche Age at menopause Pregnancy complications Menopause-associated disorders	None	Selection 3/4 Comparability 1/2 Exposure 3/3 Sum 7/9
Schweizer 2004[13]	Retrospective cohort study	Cases: 8 confirmed CD women Controls: 714 women with no history of CD CD diagnosis criteria: Celiac registry or positive IgA anti-tTG and positive EMA and positive HLA-DQA1 and HLA DQB1 typing	Age at menarche	None	Selection 3/4 Comparability 1/2 Exposure 3/3 Sum 7/9
Sferlazzas 2008 [20]	Cross-sectional Case–control	Cases: 94 untreated menarcheal CD adolescents Controls: • 117 early-treated and compliant CD girls • Cases’ non-CD mothers • 280 healthy adolescents CD diagnosis criteria: serum EMA and/or tTGA positivity and small intestinal biopsy according to grade III of Marsh’s classification	Age at menarche	None	Selection 4/4 Comparability 0/2 Exposure 3/3 Sum 7/9
Sher 1994 [5]	Case–control	Cases: 68 women with CD Controls: 68 matched healthy women Age: 16–65 years CD diagnosis criteria: N/S	Age at menarche, Age at menopause Pregnancy complications Miscarriages, Infertility	None	Selection 4/4 Comparability 1/2 Exposure 2/3 Sum 7/9
Smecuol 1996 [17]	Cross-sectional Case–control	Cases: 130 women with CD (100 with recent diagnosis) Controls: 130 healthy women Age: 13–74 (cases), 14–77 (controls) CD diagnosis criteria: “clinical, laboratory and histological”	Age at menarche, Age at menopause Pregnancy complications Menstrual history, Miscarriages	None	Selection 4/4 Comparability 1/2 Exposure 2/3 Sum 7/9
Soresi 2020 [23]	Retrospective Case- Control	Cases: 56 women with CD Controls: 71 Healthy- asymptomatic women Age: 19–66 CD diagnosis criteria: N/S	Menstrual history Infertility	Age < 18 years Self-exclusion of wheat from the diet Presence of other “organic” gastrointestinal diseases Pregnancy Immune deficiency disorders	Selection 4/4 Comparability 2/2 Exposure 3/3 Sum 9/9
Tuerxuntayi 2014[14]	Cross- sectional case–control	Cases: 67 women with CD Controls: 67 healthy women Age: 18–44 years CD diagnosis criteria: ACG guidelines	Anthropometric, Medical history Age at menarche, Menstrual history Miscarriages, E_2_, PRG, FSH, LH, PRL TSH, AMH, Folate, Ferritin, Zinc, Selenium, anti-tTG	18 < age > 44 Diagnosed and treated with a gluten-free diet or a special diet (vegetarian, dairy-free), Smoking, Pregnancy Oral contraceptive taking history in the last 6 months Gynecological and endocrine relevant surgical history Cytotoxic drugs history Pelvic radiotherapy history Diagnosed or potential PCOS Autoimmune and inflammatory diseases history, Genetic disorders, Hyperprolactinemia or other relevant diseases, Severe somatic diseases (liver, lung, or kidney diseases) Diagnosed or potential malignancies Gynecological organic diseases	Selection 4/4 Comparability 2/2 Exposure 3/3 Sum 9/9

*ACG* College of Gastroenterology, *AFC* Antral Follicle Count, *AMH* Anti-Müllerian Hormone, *AUB* Abnormal Uterine Bleeding, *BMI* Body Mass Index, *CD* Celiac Disease, *E*_*2*_ Estradiol, *EMA* Endomysial Antibodies, *ESPGHAN* European Society for Paediatric Gastroenterology Hepatology and Nutrition, *FSH* Follicle Stimulating Hormone, *GFD* Gluten-Free Diet, *ICD 10* International Classification of Diseases (K90.0: ICD10 for celiac disease), *IVF* in vitro fertilization, *LH* Luteinizing Hormone, *N/S* not specified, *PCOS* Polycystic Ovary Syndrome, *PRG* Progesterone, *PRL* Prolactin, *TSH* Thyroid Stimulating Hormone, *tTG* tissue transglutaminase

### Celiac disease and the menstrual cycle

#### Mean age of menarche

**Fig. 2:**
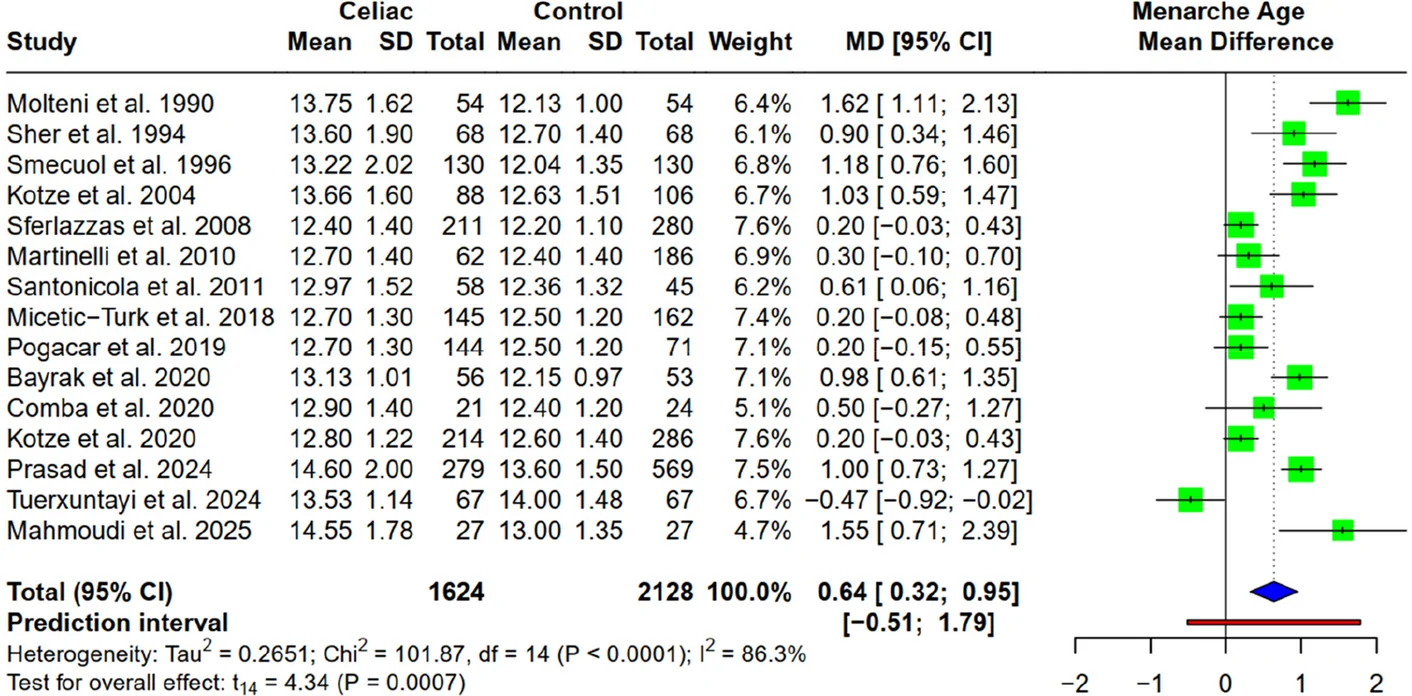
Menarche - Celiac vs. controls

Fifteen studies [5, 6, 8, 9, 14, 17, 19, 20, 25, 27, 29–33] comprising 1,624 women with CD and 2,128 controls reported mean age at menarche (Fig. 2). Women with CD experienced menarche significantly later than controls (pooled MD: 0.64 years, 95% CI: 0.32–0.95, p < 0.001). Between-study heterogeneity was considerable (I² = 86.3%). Individual study estimates ranged from −0.47 years (95% CI: −0.92 to −0.02) to 1.62 years (95% CI: 1.11–2.13), and only one study showed earlier menarche in girls with CD than in healthy girls.

##### Effect of gluten-free diet

**Fig. 3:**
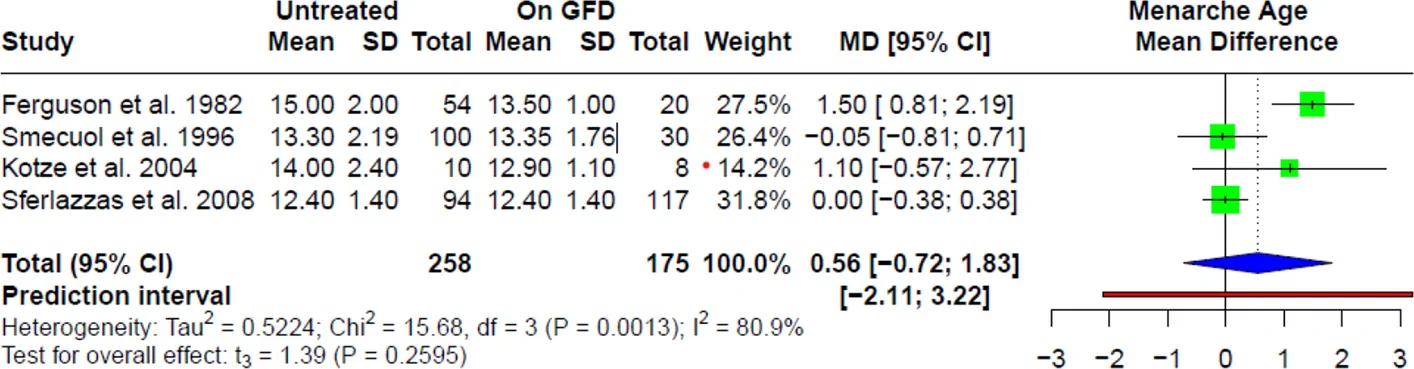
Gluten-free diet on Menarche - Untreated vs. treated CD patients

Six studies [4, 9, 13, 17, 19, 20] assessed the effects of GFD on menstrual characteristics. Only data on the mean age at menarche were available from more than three studies for quantitative synthesis. Two studies [9, 13] were excluded because it was unclear whether the participants had undergone a diet before menarche. Age at menarche was not significantly delayed in girls not adherent to gluten-free diet in the age of menarche compared to adherent girls (Pooled MD: 0.56 years, 95% CI: −0.72–1.83, p = 0.260). Between-study heterogeneity was considerable (I.^2^ = 80.9%) (Fig. 3). This analysis should be interpreted with caution, as in most studies, the timing of diet initiation in relation to menarche was not clear. Therefore, it is not possible to determine whether adherence to a gluten-free diet preceded or followed pubertal onset; therefore, no causal relationship can be established. This limitation restricts any firm conclusions regarding a potential protective effect of the gluten-free diet on pubertal timing.

#### Mean age of menopause

**Fig. 4:**
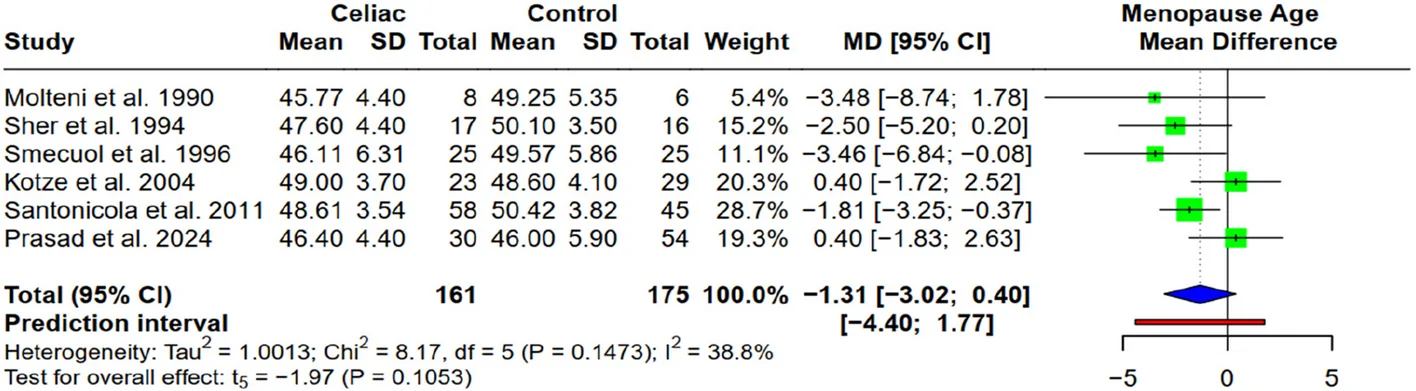
Menopause - Celiac vs. controls

Seven studies [5, 6, 9, 17, 19, 29, 33] reported the mean age at menopause, and one study was excluded from the analysis due to the inability to determine the sample size of menopausal women [29]. Six studies with 161 women with CD and 175 controls were included in the analysis (Fig. 4). Women with CD experienced menopause earlier than controls, although the difference was not statistically significant (pooled MD: −1.31 years, 95% CI: −3.02–0.40, p = 0.105). Heterogeneity was moderate (I² = 38.8%).

#### Abnormal uterine bleeding

**Fig. 5:**
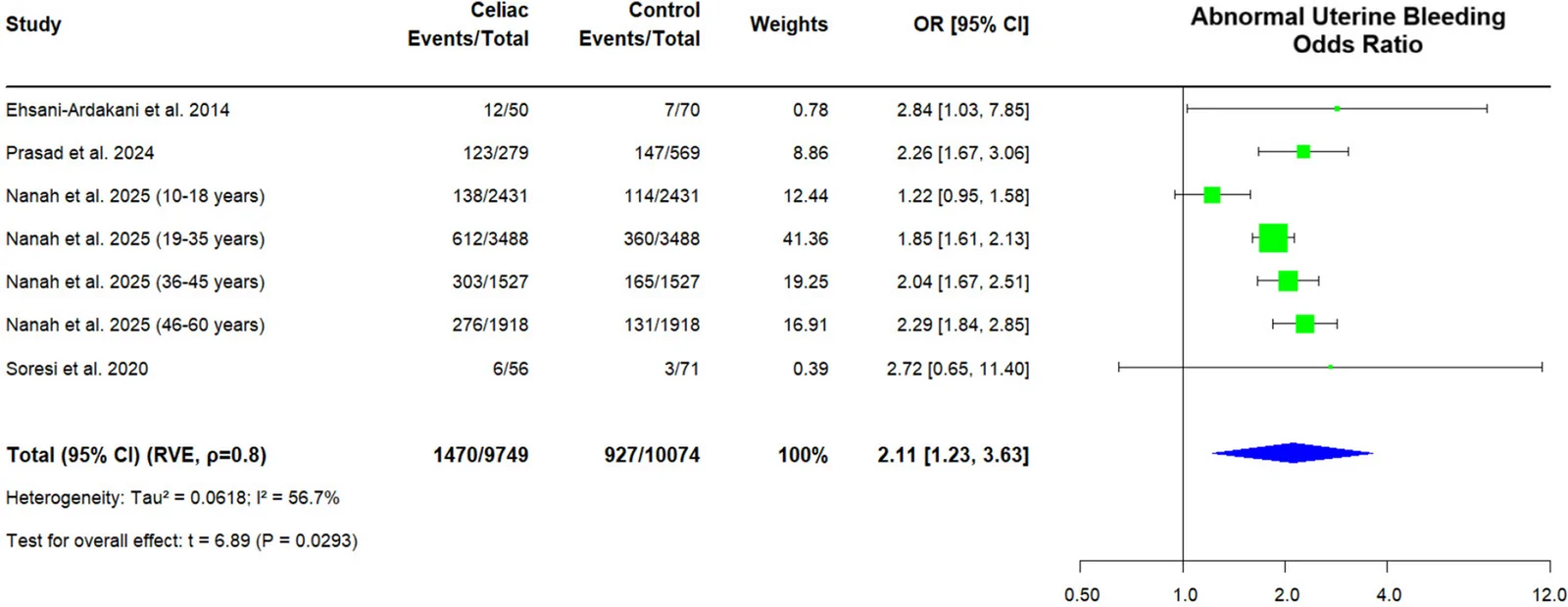
Abnormal Uterine Bleeding – Celiac vs. controls

Four studies [22, 23, 28, 33] with seven effect sizes (three studies with single estimates and one study with four age-stratified estimates) were included. A total of 9749 women with CD and 10,074 controls were included in the study (Fig. 5). Women with CD had significantly higher odds of abnormal uterine bleeding compared with controls (pooled OR: 2.11, 95% CI: [1.23–3.63], p < 0.05; RVE with ρ = 0.8). Between-study heterogeneity was substantial (I² = 56.7%).

#### Amenorrhea

**Fig. 6:**
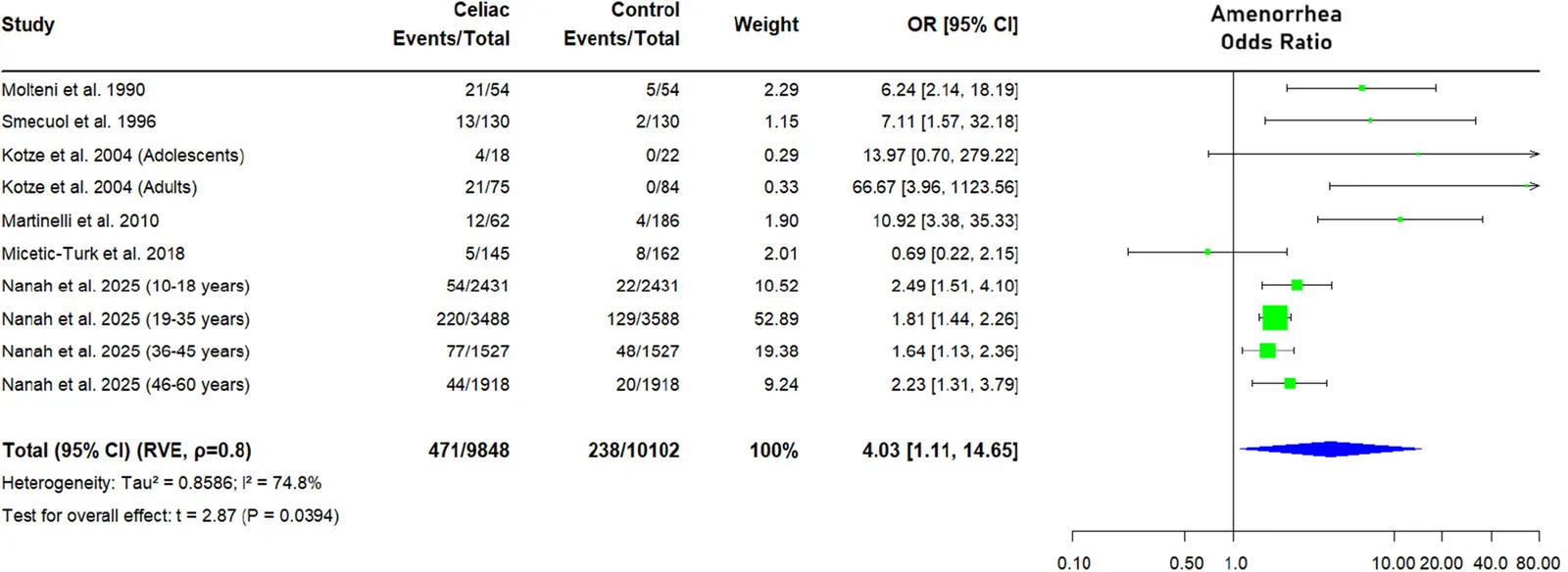
Amenorrhea - Celiac vs. controls

Six studies [6, 8, 17, 19, 22, 31] with 10 effect sizes (including one study with four age-stratified estimates and one with two age-stratified estimates) comprising 9,848 women with CD and 10,102 controls reported amenorrhea (Fig. 6). One study with zero events in both groups was excluded [23]. Women with CD had significantly higher odds of amenorrhea compared with controls (pooled OR: 4.03, 95% CI: 1.11–14.65, p = 0.039; RVE with ρ = 0.8). Between-study heterogeneity was substantial (I² = 74.8%). The individual study ORs ranged from 0.69 (95% CI: 0.22–2.15) to 66.67 (95% CI: 3.96–1123.50). The wide confidence intervals reflect the rarity of amenorrhea in some studies.

### Celiac disease and hormonal markers

#### Follicle-Stimulating Hormone (FSH)

**Fig. 7:**
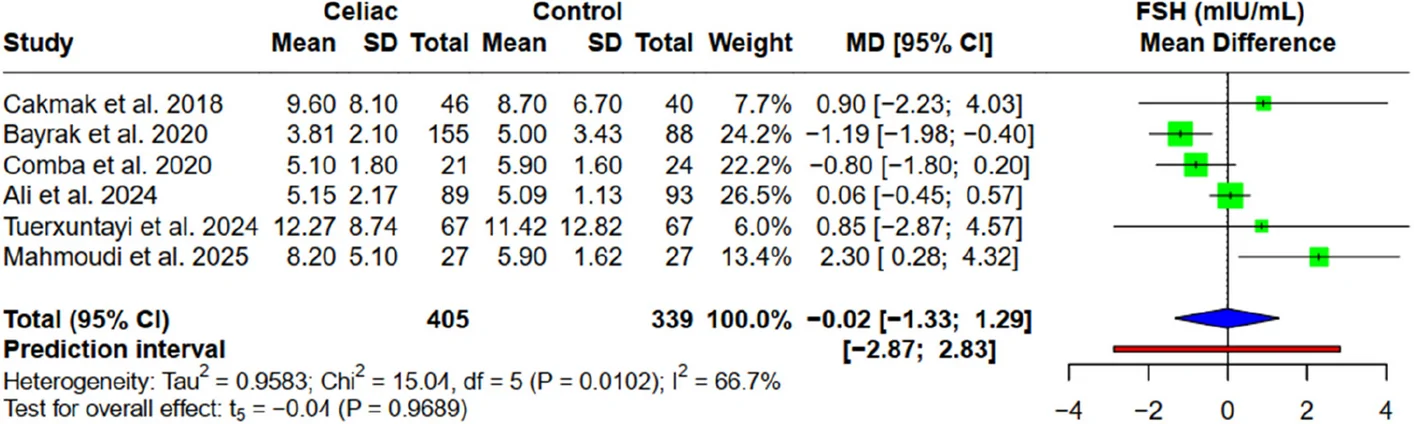
FSH - Celiac vs. controls

Six studies [14, 24, 25, 28, 50, 51] with 405 women with CD and 339 controls reported FSH concentrations (Fig. 7). No significant difference was observed between the groups (pooled MD: −0.02mIU/mL, 95% CI: −1.33–1.29, p = 0.969). Heterogeneity was substantial (I² = 66.7%).

#### Luteinizing Hormone (LH)

**Fig. 8:**
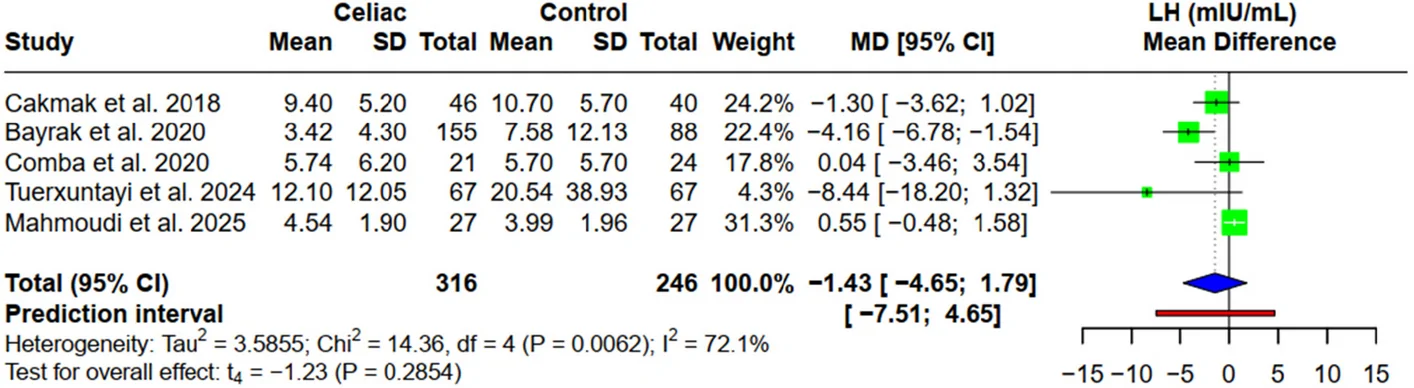
LH - Celiac vs. controls

Five studies [14, 25–27, 30] with 316 women with CD and 246 controls reported LH concentrations (Fig. 8). Women with CD showed no statistically significant lower LH concentrations than controls (pooled MD: −1.43mIU/mL, 95% CI: −4.65–1.79, p = 0.285). Heterogeneity was substantial (I² = 72.1%).

#### Estradiol (E₂)

**Fig. 9:**
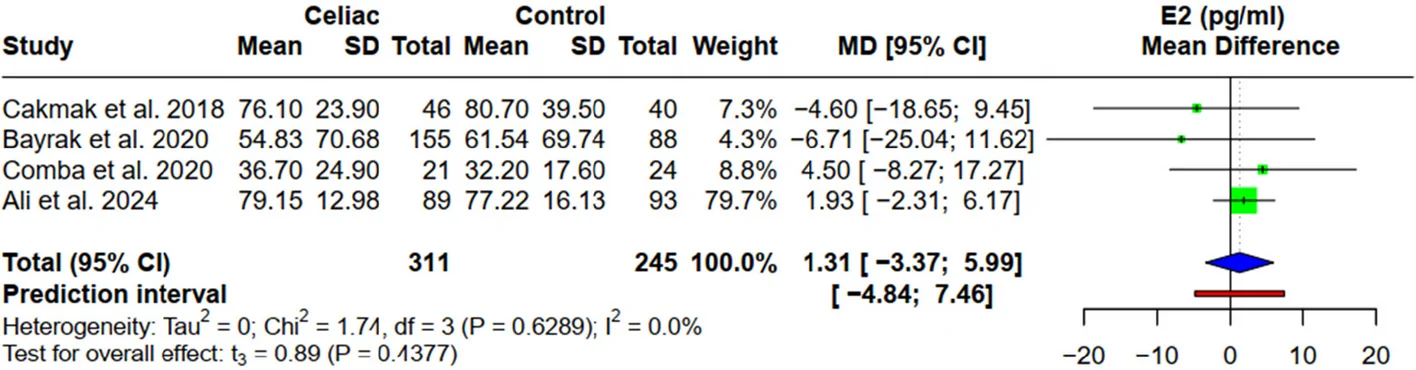
E_2_ – Celiac vs. controls

Four studies [24–27] with 311 women with CD and 245 controls reported E₂ concentrations (Fig. 9). No significant difference was observed (pooled MD: 1.31 pg/mL, 95% CI: −3.37–5.99, p = 0.438). Heterogeneity was absent (I² = 0.0%). However, given the small number of studies (*n* = 4) and small sample size (< 500), this finding should be interpreted cautiously as the statistical power to detect heterogeneity is limited. One study with highly skewed data was excluded [14].

#### Anti-Müllerian Hormone (AMH)

**Fig. 10:**
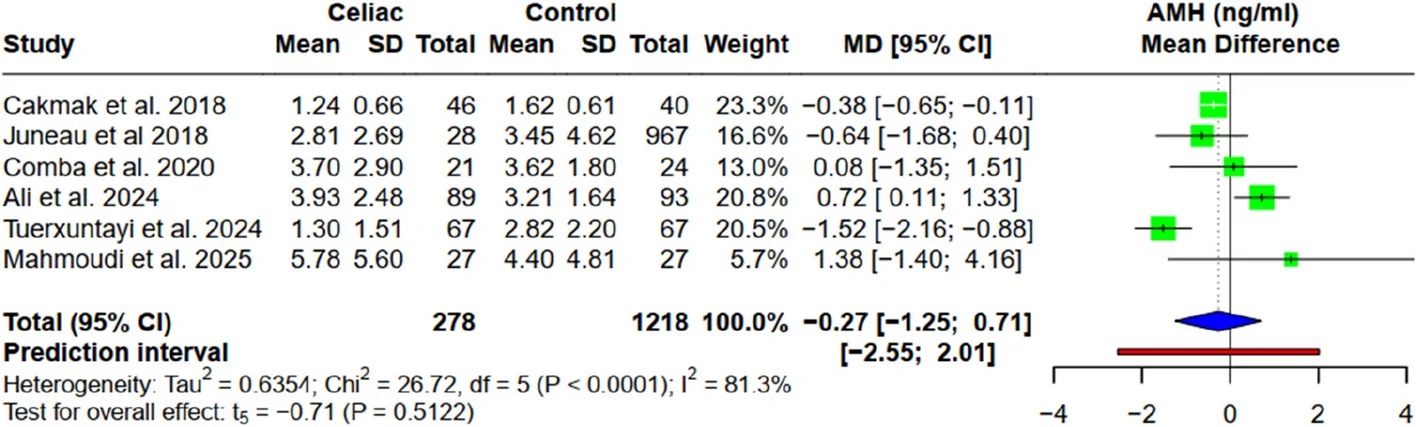
AMH – Celiac vs. controls

Six studies [14, 15, 24, 26, 27, 30] with 278 women with CD and 1218 controls reported AMH concentrations (Fig. 10). No statistically significant difference in AMH concentrations was found between the groups (pooled MD: −0.27 ng/mL, 95% CI: −1.25–0.71, p = 0.512). Heterogeneity was considerable (I² = 81.3%).

#### Prolactin (PRL)

**Fig. 11:**
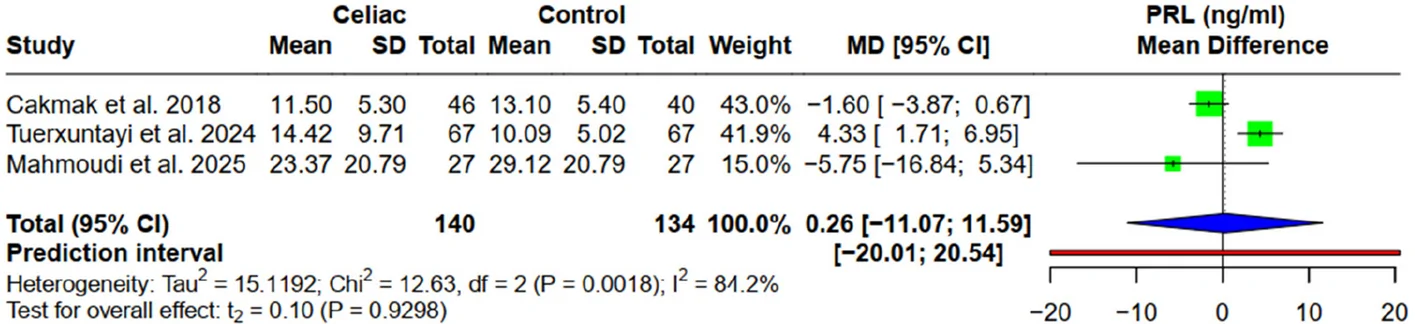
PRL - Celiac vs. controls

Three studies [14, 26, 30] with 140 women with CD and 134 controls reported prolactin concentrations (Fig. 11). No significant difference was observed (pooled MD: 0.26 ng/mL, 95% CI: −11.07–11.59, p = 0.930). Heterogeneity was considerable (I² = 84.2%).

### Subgroup and sensitivity analysis

Subgroup analyses were conducted to restrict the effects of potential confounding factors. Thus, given the potential differences in hormonal profiles between adolescents and adults, we conducted sensitivity analyses for hormonal outcomes by excluding studies that included only adolescent populations [25, 27]. This analysis was performed for FSH, LH, and AMH concentrations, where such studies existed. It was not conducted for E_2_, as there were not enough studies for quantitative synthesis in subgroups (n = 2 each), or for prolactin, which included no adolescent-only studies. These sensitivity analyses demonstrate that hormonal findings may not be driven by age-related differences and remain consistent when restricted to the rest of the studies, which only included adult populations. The relevant forest plots are included in the Supplementary Material. Regarding the effect of gluten free diet on the outcomes, only data referring to GFD on menarche were available, as data on time under diet, compliance rates, and nutritional status at menopausal age were scarce among the studies. An analysis of Tanner stages between the populations was also impossible, as only one study provided relevant data [25]. Additionally, a sensitivity analysis was performed excluding studies with NOS < 7 (Ferguson et al., 1982, NOS = 5 and Ali et al., 2024, NOS = 6). For mean age at menarche, exclusion of Ferguson et al. substantially reduced heterogeneity (I^2^ 80.9% to 0.0%), while the pooled estimate remained non-significant and consistent with the primary analysis. For FSH, AMH, and E_2_, exclusion of Ali et al. did not alter either pooled estimates or heterogeneity. These analyses confirm that results are not driven by lower- quality studies. Notably, after excluding of the large registry-based study (Nanah et al., 2025) [22], AUB remained significant (OR: 2.32, 95% CI: 1.19–4.52, I^2^ = 0%), while amenorrhea showed a consistent direction but did not reach statistical significance (OR: 5.20, 95% CI: 0.92–29.25, p = 0.057); these results are presented in the Supplementary Material. A sensitivity analysis excluding studies that required conversion of medians to means was also performed for FSH, LH, and AMH. The pooled estimates remained non-significant across all three outcomes. For prolactin, insufficient studies with originally reported means precluded a formal sensitivity analysis, and the results should be interpreted with appropriate caution. Notably, heterogeneity for AMH was substantially reduced upon excluding converted studies (I^2^ = 81.3% to 0.0%), suggesting that distributional differences among converted studies may partly account for the between-study variability observed in the primary analysis. The corresponding Forest plots are provided in the Supplementary Material.

### Leave-one-out analysis

A leave-one-out sensitivity analysis was conducted for all outcomes, excluding each study consecutively, to investigate the existence of any significant effect on pooled estimates (Supplementary Material). The results were robust, with no single study exerting undue influence. The non-significant findings for mean age at menopause, FSH, LH, E_2_, AMH, prolactin, and mean age at menopause for treated and untreated CD remained non-significant across all iterations, whereas the significant findings for mean age at menarche, abnormal uterine bleeding, and amenorrhea remained robust. For outcomes analyzed using the RVE (abnormal uterine bleeding and amenorrhea), we assessed the sensitivity to the assumed within-study correlation. The results were highly consistent across different correlation assumptions, demonstrating that the findings were not sensitive to the specific correlation assumption used.

### Publication bias assessment

Funnel plot asymmetry and Egger’s regression were used to assess publication bias for each outcome, although the statistical power of these tests may not be high enough to detect real asymmetry in small samples. With a visual interpretation of the funnel plots (Supplementary Material), Egger’s regression test, and the trim-and-fill method, we concluded that no increased risk of publication bias was observed for nine of the ten outcomes. In contrast, evidence of publication bias was identified for abnormal uterine bleeding (funnel plot asymmetry, Egger’s test p < 0.05). After trim-and-fill adjustment (although not fully valid under robust variance estimation), the pooled effect estimate was attenuated but remained in the same direction (OR: 1.95, 95% CI: 1.08–3.55), indicating that the overall conclusion remained robust.

## Discussion

Our results support the impact of CD on fertility lifespan and menstrual irregularity. Menarche was significantly delayed in women with CD, and according to the limited bibliography, adherence to a gluten-free diet did not change this correlation. This result may reflect the timing of dietary intervention, as no minimum time period on diet before menarche was always held between the studies, or imply that even early life exposure to malnutrition, chronic inflammation, or autoimmune activity may exert a lasting effect on pubertal timing that is not fully reversible with later dietary treatments. In any case, this finding must be examined with caution, as the included studies lack a thorough design referring to the timing of GFD initiation, compliance, and remission rate through antibody monitoring and may differ in terms of disease severity at diagnosis and treatment initiation. A tendency toward a younger menopausal age was also observed, although it was not statistically significant. However, two of the six studies examining menopausal age reported a significantly younger menopausal age in women with celiac disease, and none of the included studies suggested a later menopausal age. The tendency toward lower AMH levels observed in patients with celiac disease, combined with the small number of menopausal women included in the current literature, indicates the need for further well-designed studies to clarify the potential impact of celiac disease on ovarian aging and menopausal timing. Moreover, women with CD were found to be at a greater risk of experiencing amenorrhea and abnormal bleeding compared to control populations. In addition, our results confirm that this impact is not represented by differences in hormonal markers. Hormonal markers were comparable between the groups, although a tendency for lower AMH concentrations was observed in patients with CD.

The effect of CD on the above-mentioned menstrual and hormonal outcomes can be interpreted based on some theories. The main axes are malnutrition, micronutrient deficiencies, and systematic chronic inflammation. Both Selenium and Zinc seem to play vital roles in the mechanisms of the variant reproductive systems, affecting the synthesis of gonadotropins and sex hormones, follicle growth and maturation, as well as directly affecting endometrial proliferation [52, 53]. Micronutrient deficiency in CD has also been proven in a recent meta-analysis [14, 54]. Leptin concentrations, a hormone with a pivotal role in hypothalamic-pituitary-ovarian axis pubertal activation, were found to be significantly lower in CD compared to healthy children [55]. This difference was significantly related to GFD but not to the Marsh score intestinal histologic lesion grade. Data also support the negative impact of chronic illness on menarche and menstrual patterns [56], while a higher prevalence of premature ovarian insufficiency has also been observed in women with autoimmune diseases [40, 57]. Differentiation of the gut microbiota has also been associated with hormonal changes and malfunctions [58, 59].

A limitation to be acknowledged is that several studies reported hormonal markers with interquartile ranges as medians, necessitating conversion to means and standard deviations [14, 15, 26]. However, hormonal markers, such as AMH and prolactin, frequently exhibit non-normal distributions, and such conversions may introduce bias in the pooled estimates. Although one highly skewed study was excluded, conversions may affect the precision of the hormonal outcome analyses. A sensitivity analysis excluding all mathematically converted studies was performed for FSH, LH, and AMH; the results remained non-significant and consistent with the primary analyses. Notably, heterogeneity for AMH was substantially reduced upon exclusion of converted studies (I^2^ = 81.3% to 0.0%), identifying data conversion as a significant source of between-study heterogeneity for this outcome.

In addition to data conversion, several factors contribute to the observed heterogeneity among the included studies. Significant heterogeneity was observed for several outcomes, including age at menarche (I^2^ = 86.3%), amenorrhea (I^2^ = 74.8%), AMH (I^2^ = 81.3%), prolactin (I^2^ = 84.2%), and LH (I^2^ = 72.1%), reflecting important differences in study design and methodology. Regarding hormonal markers, although most studies collected blood samples during the early follicular phase, laboratory methods and the exact timing were not identical across studies. For example, one study [26] measured AMH using an ELISA kit, while FSH, LH, E_2_, and prolactin were analyzed by chemiluminescent microparticle immunoassay, another study [30] used different commercial kits for AMH and the other hormones and some studies did not mention the exact kits used [24, 25, 27]. An additional source of heterogeneity is the wide variation in study designs, ranging from small case–control studies with limited numbers of participants [6, 9, 30, 31] to larger [12, 19] and registry-based analyses [22], which may disproportionately influence the pooled estimates, as evidenced by the sensitivity analysis excluding Nanah et al., which substantially reduced heterogeneity for AUB.

Additional sources of heterogeneity include differences in the method of menstrual characteristics collection, ranging from self-reported questionnaires [8, 31, 33] to physician-completed histories [17, 19, 28] and registry-based data [22]. Moreover, studies differed in population characteristics, including adolescent populations [25, 27], adult reproductive-age women [14, 24, 26, 30], and mixed-age groups, including treated and untreated patients. To assess this discrepancy, a subgroup analysis referring to hormonal markers, excluding studies [25, 27] with adolescent populations, was conducted, and no significant effect on the results was observed. Different exclusion criteria, which can vary substantially, as shown in the table should above, also be considered.

To investigate potential sources of heterogeneity, several sensitivity and subgroup analyses were conducted, including restriction to adult populations excluding adolescent-only studies, exclusion of mathematically converted data, exclusion of studies with NOS < 7, and exclusion of the large registry-based study (Nanah et al., 2025). Although these analyses influenced heterogeneity estimates for certain outcomes, as discussed above, the pooled estimates remained consistent with the primary analyses, supporting the overall robustness of our findings.

Unfortunately, data referring to Tanner stage, time on gluten-free diet, and compliance with the diet via antibody monitoring were scarce among the studies, and no relevant analysis could be carried out. Dietary status analysis referring to each outcome could only be conducted for the age of menarche according to the provided data. The diagnostic criteria for CD varied from histology-confirmed cases to serology-based identification of undiagnosed disease, and not all of the included studies screened the control group for CD antibodies [13, 23]*.*

Despite the growing body of evidence, the available data remain limited by small sample sizes, heterogeneous methodologies, and observational study designs. Larger, well-designed prospective studies with standardized diagnostic criteria and uniform assessments of reproductive outcomes are needed to provide more robust and conclusive evidence. This is particularly important given the clinical relevance of the findings, as CD frequently manifests in childhood and adolescence and may affect the critical window of reproductive development. Improving the quality of evidence in this field is essential to better inform clinical practice and preventive strategies aimed at protecting the long-term reproductive health of women affected by this condition.

## Conclusion

Our analysis indicates that women with celiac disease experience menstrual cycle disturbances more frequently and tend to have a later age at menarche compared with healthy controls. However, no statistically significant differences were observed between the two groups in hormonal parameters or markers of ovarian reserve.

These findings suggest that celiac disease, possibly through autoimmune-mediated mechanisms or through malabsorption and micronutrient deficiencies, may influence ovarian function in a manner that disrupts menstrual regularity and cycle duration without producing detectable alterations in commonly used hormonal biomarkers.

## Supplementary Information

Supplementary Material 1.

## Data Availability

As this is a meta-analysis of published studies, data were extracted from the included published studies. Our extracted data of each study are demonstrated in the relevant forest plot for each outcome. The datasets (excel spreadsheets) analyzed during the current study are available from the corresponding author on reasonable request.
