# Supplementary material for: Association of celiac disease with menstrual and hormonal disturbances: a systematic review and meta-analysis
Source: Reprod Biol Endocrinol. 2026 Jun 22;24:82. doi: 10.1186/s12958-026-01572-7 (PMC13555964; doi:10.1186/s12958-026-01572-7)
Supplement: Supplementary file 1 [file 12958_2026_1572_MOESM1_ESM.docx]

**Supplementary Material**

**Leave One out analysis**

1. Mean age of menarche

2. Meanage of menopause

3. FSH

4. LH

5. E_2_

6. AMH

7. PRL

8. Mean age of menarche for treated vs. untreated

**Publication bias (Funnel plots)**

1. Mean age of menarche

2. Meanage of menopause

3. FSH

4. LH

5. E_2_

6. AMH

7. PRL

8. Mean age of menarche for treated vs untreated

**Subgroup analysis**

1. FSH adults

2. LH adults

3. AMH adults

**Sensitivity analysis excluding converted data from median to mean**

1. FSH

2. LH

3. AMH

**Sensitivity analysis excluding study of Nanah et al. 2025**

1. Abnormal Uterine Bleeding

2. Amenohhrea

**Sensitivity analysis excluding studies with NOS <7 on ROB assessment**

1. Menarche on diet excluding Ferguson et al. 1982

2. FSH excluding Ali et al. 2024

3. AMH excluding Ali et al. 2024

4. E2 excluding Ali et al. 2024

**Risk of Bias assessment**
